# Exercise Preferences, Barriers, Motivators, Facilitators, and Perceived Benefits in Adults With Brain Tumours—A Systematic Review

**DOI:** 10.1002/cam4.71731

**Published:** 2026-03-31

**Authors:** Max E. Walker, Meg A. Doohan, Georgia K. B. Halkett, David Mizrahi, Lisa Simmons, Caroline V. Robertson, Justin J. Chapman

**Affiliations:** ^1^ Centre for Mental Health, School of Pharmacy and Medical Sciences Griffith University Brisbane Australia; ^2^ Curtin School of Nursing, Faculty of Health Sciences Curtin University Perth Australia; ^3^ Curtin Medical Research Institute, Faculty of Health Sciences Curtin University Perth Australia; ^4^ The Daffodil Centre, the University of Sydney, A Joint Venture With Cancer Council NSW Sydney Australia; ^5^ College of Medicine and Dentistry James Cook University Townsville Australia; ^6^ Queensland Centre for Mental Health Research Brisbane Australia; ^7^ Addictions and Mental Health Service, Metro South Health Brisbane Australia; ^8^ Equally Well, Charles Sturt University New South Wales Australia

**Keywords:** barriers, brain tumours, facilitators, perceived benefits, physical activity, preferences

## Abstract

**Introduction:**

Adults with brain tumours face a poor prognosis, and physical and mental impairments reduce quality of life. Exercise can improve outcomes for this population; however, uptake and adherence remain low. Understanding exercise preferences, barriers, motivators, facilitators, and perceived benefits is crucial to optimising engagement.

**Methods:**

A systematic search of Embase, MEDLINE, Scopus, CINAHL Complete, and the Nursing and Allied Health Database (2015–2025) was conducted. Studies were eligible if they included adults (≥ 18 years) with brain tumours and reported original qualitative or quantitative data. Screening, data extraction, and quality appraisal (Mixed Methods Appraisal Tool, 2018 version) were performed by two authors, with discrepancies resolved by a third. Data were synthesised narratively due to heterogeneity.

**Results:**

Seven studies involving 163 participants were included, with mean ages of 48–63.5 years and 44.8% males. Most studies included individuals diagnosed with glioblastoma, glioma, or oligodendroglioma. Exercise preferences were reported in 5/7 studies. Participants preferred flexible, individualised exercise programs with varied delivery modes and session durations. Walking was the most commonly preferred activity in 2/7 studies (48%–56% of participants); 1/7 study reported 65% chose multiple activities, including cycling, swimming, and running. Barriers were identified in 5/7 studies, including symptom burden, cognitive impairment, treatment‐related side effects, and psychological and external factors. Facilitators and motivators were reported in 3/7 studies and included support from carers and healthcare providers, structural enablers, and intrinsic motivations. Perceived benefits were identified in 5/7 studies, including improved physical function, energy, quality of life, well‐being, self‐efficacy, and social engagement.

**Conclusions:**

Exercise interventions for adults with brain tumours should be flexible, individualised, and supported by carers and healthcare providers. Moderate aerobic training is preferred, while resistance training remains underexplored. Addressing barriers and providing structured support may enhance uptake and adherence. Further research is needed to establish optimal program characteristics across disease stages.

## 1 Introduction

Globally, an estimated 321,476 new cases of brain tumours were reported in 2022 [1]. It has been predicted that if incidence rates remain stable, there will be 474,000 new cases by the year 2045, which is a 47% increase from 2022 [2]. Once diagnosed, the prognosis for malignant brain tumours is generally poor, with the five‐year survival rate ranging from 32%–38% for oligodendroglioma, 20%–38% for diffuse and anaplastic astrocytoma, and 4%–17% for glioblastoma [3]. In 2022, brain tumours accounted for approximately 248,305 deaths worldwide [1]. This statistic places brain tumours as the 12th leading cause of cancer‐related deaths worldwide [1]. The World Health Organisation's (WHO) classification of brain tumours was updated in 2016 and 2021 to incorporate molecular profiling, which is now a key indicator of diagnosis and prognosis. The tumour microenvironment has become a crucial aspect of cancer care, and the interaction between the tumour microenvironment, treatments, and lifestyle factors such as exercise is shaping emerging directions in both oncological research and clinical models of care [4].

Current treatment options for primary and secondary brain tumours are associated with numerous complications and side effects [5]. Surgical interventions, dependent on resection extent and tumour location, may cause language, motor, and cognitive impairments (i.e., working memory, executive functioning, and attention) [6, 7]. Radiotherapy can adversely affect the brain's plasticity and repair processes [8]. Post‐radiation therapy, fatigue, disturbed sleep, and depression are commonly reported, often affecting the ability to work, maintain relationships, perform daily activities, and quality of life [9]. Chemotherapy further contributes to infection risks, hair loss, mouth sores, and gastrointestinal side effects, including loss of appetite, nausea, and vomiting [10]. Additionally, brain tumours impair muscle strength, balance, and gait, and functional capacity [11, 12, 13]. Alongside these physical limitations, individuals often experience psychological challenges such as a sense of altered identity, social withdrawal, and reduced relational closeness [14]. Consequently, physical activity levels in this population remain low, with only 22%–41% of individuals meeting guidelines during or post‐treatment compared to more common cancers (e.g., breast, colorectal, prostate—54%) [15]. These levels are substantially lower than those observed in the general adult population, where approximately 69% meet recommended physical activity guidelines [16]. These challenges are often compounded by loss of independence and inability to drive due to cognitive and functional decline [17, 18].

Given this significant symptom burden and limitations of current treatment options, there is a strong rationale for implementing exercise as a supportive care intervention [19, 20]. Individuals with brain tumours have reported improvements in fatigue, depression, drowsiness, and concern with well‐being after participating in a 12‐week exercise program [21]. Recent evidence in non‐pharmacological supportive care interventions—including exercise—has highlighted promising effects on patient‐reported outcomes such as quality of life, psychological wellbeing, fatigue, and cognitive function in individuals with brain tumours [22]. Exercise has been shown to modulate immune cells, support angiogenesis and vascular structure, reshape immune and metabolic networks, regulate the immune microenvironment of the tumour and the whole body—ultimately impacting the entire immune system [4]. Encouragingly, a supervised aerobic and resistance exercise intervention for individuals with primary brain tumours undertaking adjuvant chemoradiotherapy reported no seizures during clinical testing or moderate‐intensity exercise, suggesting that exercise is safe under professional supervision [23]. Understanding the feasibility of exercise participation, including adherence, engagement, and long‐term sustainability among individuals with brain tumours, could help inform the design of exercise interventions for this group.

Exercise engagement among individuals with brain tumour varies across the disease trajectory, with individuals' ability and willingness to participate influenced by treatment stage and symptom burden [24]. A recent review of individuals with brain tumours suggested that the effectiveness of exercise interventions may be improved when tailored to participants' needs [15]. Understanding this, in a study with individuals diagnosed with glioblastoma, only half of the recruited participants were willing or able to initiate the program, complete it, or adhere to minimum dose requirements [23]. Additionally, feasibility was dependent on health status and treatment tolerance, with compliance to the exercise prescription ranging from 24%–83% despite all participants receiving similar treatments for high‐grade glioma. The uptake of and adherence to future exercise interventions may be more successful if they are congruent with individuals' interests, preferences, and treatment plan [25].

Therefore, this systematic review aimed to synthesise the current evidence on exercise preferences, barriers, motivators, facilitators, and perceived benefits among adults diagnosed with brain tumours.

## 2 Methods

This systematic review was conducted following the Preferred Reporting Items for Systematic Reviews and Meta‐Analysis (PRISMA) guidelines [26] and was registered in the international prospective register of systematic reviews (PROSPERO ID Number: CRD420251037673).

### 2.1 Search Strategy

A systematic search was conducted across Embase, MEDLINE (Ovid), Scopus, CINAHL Complete (EBSCOhost), and Nursing and Allied Health Database (ProQuest) on 24 March 2025 by the primary author (MW). The search strategy was developed in collaboration with a scientific librarian, ensuring the use of appropriate search terms and subject headings for each database (Table S1). Database‐specific limiters were applied to identify publications in English and publications within the last 10 years (2015–2025) to ensure that findings were both current and translationally relevant.

### 2.2 Eligibility Criteria

Eligible studies recruited adult participants (≥ 18 years old) who were either currently or had previously been diagnosed with primary or secondary benign or malignant brain tumours. Studies of any design and quality were included, provided they presented original qualitative or quantitative data on participants' exercise preferences, barriers, motivators, facilitators, and perceived benefits of exercise. Data collection methods included surveys, semi‐structured interviews, and focus groups. Studies were excluded if they involved only paediatric or adolescent populations. Reviews and meta‐analyses were also excluded.

### 2.3 Study Selection

The screening process was conducted by two authors (MW, MD), using Covidence (Veritas Health Innovation, 2025). If a title and abstract met the study eligibility criteria, then the full text of the study was obtained for further review. Any conflicts were resolved by the senior author (JC) following discussion.

### 2.4 Quality Appraisal

The Mixed Method Appraisal Tool (MMAT, version 2018) was used to assess the quality and risk of bias of the included research [27]. This tool can be used to appraise the quality of empirical qualitative, quantitative, and mixed methods studies. The primary author (MW) conducted the quality assessment of the included literature, and the results were reviewed by the second and senior author (MD, JC).

### 2.5 Data Extraction and Statistical Analysis

Data extraction was conducted by two authors (MW, MD) using a pre‐piloted Microsoft Excel spreadsheet. Details on study design, sample size, population characteristics (i.e., age, brain tumour subtype, treatment type, etc.), and key findings about exercise preferences, barriers, motivators, facilitators, and perceived benefits were recorded. Although some included studies recruited patient‐carer dyads, only data provided by individuals with brain tumours were extracted and synthesised. Carer responses were not included in the analysis. A narrative synthesis approach was used to integrate the studies, group similar findings, describe patterns and differences across studies, and identify areas where results converged or conflicted. A meta‐analysis was not conducted due to substantial heterogeneity in study designs, outcome measures, and reporting of exercise preferences, barriers, facilitators, motivators, and perceived benefits across studies.

## 3 Results

### 3.1 Study Selection

A total of 6321 records were identified from the systematic search: Scopus (*n* = 3116); Embase (*n* = 2247); MEDLINE (*n* = 575); CINAHL (*n* = 186); and Nursing and Allied Health Database (*n* = 197), respectively. After removing 1870 duplicates, 4451 article titles and abstracts were screened, and 25 records remained for full‐text screening. Of these, 18 records were excluded due to reporting irrelevant outcomes (*n* = 6), being conference proceedings (*n* = 8), or being conducted with people under 18 years old (*n* = 4). After the screening process was complete, seven studies were deemed eligible to be included in this systematic review. Figure S1 provides the PRISMA diagram for the article screening process.

### 3.2 Demographic Characteristics

A total of seven studies involving 163 participants were included in the synthesis.

Sample sizes ranged from 5 to 51 participants. The mean age of participants across studies ranged from 48 to 63.5 years. Six studies reported sex, with 44.8% of participants identified as male [28, 29, 30, 31, 32, 33]. Six studies specified cancer type, the most common of which were diagnosed with glioblastoma (*n* = 51), followed by glioma (*n* = 21), and then oligodendroglioma (*n* = 16) [28, 29, 30, 31, 33, 34]. The remaining participants had other brain cancer diagnoses (*n =* 48) or unspecified types (*n =* 31). A summary of the demographic characteristics can be found in Table S2.

### 3.3 Quality Assessment

The quality appraisal is reported in Table S3. When the outcomes of interest for this review (assessing preferences, barriers, motivators, facilitators, and perceived benefits of exercise) did not align with the primary outcome for the included study, the quality appraisal related to the relevant evidence being considered rather than the study design. This aligns with the Cochrane Handbook for Systematic Reviews of Interventions, which states that, prior to appraisal, reviewers must “select which specific results from the included trials to assess” and that “an approach that focuses on the main outcomes of the review may be the most appropriate approach” [35]. For example, because Culos‐Reed et al. [28] reported exercise preferences at baseline, the intervention was not relevant to the review question, so the quality of evidence was appraised as a cross‐sectional design. Similarly, Piil et al. [33] was a mixed methods study; however, because only the qualitative data were relevant to the review, the study was appraised as a qualitative study. Evidence from the included studies was appraised as cross‐sectional, comprising four qualitative and three quantitative descriptive studies. All four qualitative studies met all the criteria for methodology and were assessed as high quality. Gehring et al. [31] and Lowe et al. [32] had a common challenge of non‐response bias. Gehring et al. [31] had low recruitment (25%) of invited participants, indicating potential risk of recruitment bias, which consequently resulted in a high risk of non‐response bias. Lowe et al. [32] provided no data on non‐participants, response rates, or demographic comparisons, preventing assessment of non‐response bias.

### 3.4 Exercise Preferences

Exercise preferences were reported in five of the seven studies. Findings showed that participants favoured adaptable, individualised exercise programs that accommodated flexible delivery modes, session structures, and personal circumstances [28, 29, 30, 31, 32]. Participants reported a desire for choice between online, in‐person, and hybrid mediums, and both individual and group‐based delivery modes [29, 30]. In one study, one‐on‐one sessions were preferred due to the opportunity for individualisation [29]. In terms of session structure, preferred session duration varied widely from 15 to 90, and 2–3 sessions per week at moderate intensity [29, 32].

These preferences were dependent on factors such as symptom burden, energy levels, treatment stage, and personal goals [29]. Participants reported needing flexible sessions (e.g., shorter or adaptable) to accommodate fluctuating fatigue and physical function [29]. Treatment stage also determined preferences, with those earlier in their cancer journey focusing on regaining physical function, while others prioritised maintaining routines and managing symptoms [29]. Home‐based settings were commonly preferred [28, 32]; 29% of participants preferred exercising with family and friends [32], whereas others preferred to exercise alone (44%) and unsupervised (71%) [28]. Culos‐Reed et al. [28] also reported that 56% of participants preferred to exercise during the course of treatment.

Of the two studies that assessed preferred activity type, walking was the most preferred activity type for 48%–56%, respectively [28, 32]. One study reported that among participants with stable grade II or III brain tumours, 65% chose a combination of activities including indoor/outdoor cycling, swimming, and running [31]. Finally, all 16 participants in one study preferred recreational exercise over competitive exercise (i.e., activities involving competing or ranking against others) [28]. A summary of exercise preferences reported across studies is presented in Table S4.

### 3.5 Exercise Barriers

Five studies reported barriers to exercise participation. Several barriers were identified, reflecting both physical and psychological challenges [29, 31, 32, 33, 34]. Commonly cited obstacles to exercise were symptom burden and disease progression; participants described difficulties with managing treatment‐related side effects, such as ear‐related issues in an isolated case that prevented swimming [34], and experienced physical and cognitive decline [33]. In one palliative care cohort, 65% were not interested in an exercise program, and 58% felt unable to participate in exercise due to their health [32]. Psychological barriers included reduced motivation, limited self‐discipline, fear of adverse events such as seizures, and a perceived loss of independence and control [29, 31]. Participants also reported external factors such as challenges accessing clear information, completing assessments, managing time, balancing treatment schedules, and adhering to programs [29, 31, 34]. A summary of exercise barriers reported across studies is presented in Table S4.

### 3.6 Exercise Motivators and Facilitators

Three studies reported on motivations for exercise participation, including psychosocial, relational, and structural motivators. Participants indicated that support from carers and healthcare providers encouraged both exercise initiation and adherence [29, 30]. Additionally, structural features were identified as key facilitators for exercise, such as efficient referral processes, flexibility to choose session times, scheduled appointments that reinforced accountability and routine, and accessibility via free or low‐cost sessions [29, 30]. Intrinsic motivation also played a critical role, with individuals driven by personal goals, competitiveness, and the desire for quality of life—particularly as a way of staying active and hopeful during treatment [30, 33]. Daun et al. [29] also reported that some participants were motivated by the opportunity to inspire others facing similar circumstances. A detailed summary of these barriers and facilitators, organised by cancer/treatment stage, is presented in Table S5.

### 3.7 Perceived Benefits of Exercise

Five studies reported on the perceived benefits of exercise associated with participation in the exercise intervention. Physical benefits included improved fitness, body composition, functional capacity, energy levels, and overall quality of life [29, 30, 33, 34]. Lowe et al. [32] identified that a minority of participants (10%) described exercise as a way of “being healthy.” Psychological benefits included enhanced emotional well‐being, greater motivation and confidence, a reduction in negative thoughts, a regained sense of control, and a strengthened sense of hope and personal agency [29, 30, 33, 34]. Participants cited that engaging in exercise encouraged other health‐promoting activities such as improved sleep, better nutrition, and increased engagement in daily activities [29, 30]. Additionally, social aspects contributed to perceived benefits, with participants appreciating shared experiences, mutual support, and a safe space to express challenges [29, 34]. Participants also reported that exercise improved treatment tolerance [30]. A summary of perceived benefits reported across studies is presented in Table S4.

## 4 Discussion

This systematic review aimed to synthesise the current evidence about exercise preferences, barriers, motivators, facilitators, and perceived benefits among adults diagnosed with primary or secondary brain tumours. Although there is growing evidence supporting exercise as an adjunct to treatment for brain tumours [4, 19], limited empirical research exists examining the perspectives of individuals with brain tumours. This gap aligns with broader trends in exercise oncology research, which is currently predominantly focused upon common cancers such as breast and prostate [36]. This review sought to address this gap by providing insight into the exercise‐related needs of this population.

Preferences for flexible, individually tailored exercise programs reflect the fluctuating symptom burden and treatment‐related side effects characteristic of brain tumour trajectories [29, 32]. These preferences for flexible scheduling and hybrid delivery modes align with autonomy, while reported physical improvements reflect competence. This is consistent with recent research in similar cohorts (e.g., mixed cancer and breast cancer), which suggests that individuals are more likely to adopt physical activity when they feel self‐efficacious [37]. Variability in preferences for exercise sessions may therefore be expected in brain tumour populations, given differences in individual circumstances and health‐related factors [29]. These findings underscore the need for adaptable, individual programming that allows for ongoing modulation. The preference for walking is likely due to its low cognitive and physical demands, accessibility, and perceived safety, particularly for individuals managing balance impairments and/or fatigue [28, 31, 32]. Although participants also found that other forms of aerobic exercise were acceptable. These preferences are consistent with findings from broader cancer populations, which highlight the importance of flexibility of exercise programming [38].

Resistance training was prescribed prominently in existing intervention designs [29, 30, 34]. This highlights a potential misalignment between intervention design and participant preferences, since walking was commonly identified as the most preferred activity both in this review and in broader cancer populations (i.e., mixed, breast, lung, brain, colorectal, gynaecologic, head and neck, and others) [38]. To date, resistance exercise remains a relatively unexplored area of research in brain tumour populations [39]. While resistance training is considered safe and feasible when performed under supervision [23], its lower preference among individuals with brain tumours may be the result of fatigue and lack of confidence associated with the activity [31, 32]. Future research is warranted to explore strategies that enhance the enjoyment of resistance training, such as education and the involvement of individual preferences in program design.

A preference for home‐based exercise programs may reflect a desire for greater autonomy and reduced accessibility burden [28, 32]. Gehring et al. [31] found that incorporating features known to enhance treatment fidelity and enjoyment helped overcome potential barriers such as travel distance, uncertainty, motivational problems, or time constraints. The authors noted that remote support elements—such as regular contact and guided feedback—appeared to improve adherence. Gehring et al.'s [31] study was limited to individuals with stable gliomas, which raises questions about applicability to the broader brain tumour population. Notably, this intervention focused solely on aerobic activity; however, combining it with resistance training may yield greater benefits. A review in mixed cancer populations (i.e., breast, rectal, head and neck, prostate, lung, nasopharyngeal, and others) found that combining resistance and aerobic training resulted in greater improvements in quality of life and fatigue than resistance training alone [40]. Across broader cancer populations, persistent treatment‐related side effects, lack of time, and fatigue have been identified as the most commonly reported barriers to initiating or maintaining exercise participation [41]. In individuals with brain tumours, these barriers may be compounded by disease‐specific factors such as seizures, cognitive deficits, and motor impairments [42]. In contrast, Lowe et al. [32] found that individuals in a palliative care cohort reported low interest in, or perceived ability to participate in, exercise. This may reflect concerns about safety and feasibility rather than a lack of motivation. Motivations for exercise in cancer survivors often include improving fatigue and overall physical functioning [43]. However, for individuals with brain tumours, exercise motivations may focus on preserving functional capacity and independence [19]. Taken together, these barriers and motivations highlight the need to understand how exercise interventions can be conducted safely and effectively in this population. Whether these positive outcomes can be translated to individuals with more advanced disease and without supervision warrants further investigation in future feasibility studies.

Nevertheless, the implementation of exercise interventions in brain tumour populations remains challenged by a complex interplay of physical, psychological, and logistical factors. Physical barriers such as fatigue and cognitive decline are likely amplified in brain tumour populations due to neurological involvement and treatment‐related side effects [33, 34]. Cognitive dysfunction, which is particularly prevalent in brain tumour populations, is often exacerbated by chemotherapy and radiotherapy and has been shown to interfere with rehabilitation [44]. Psychological barriers frequently mentioned were reduced motivation, fear of seizures, and lack of independence [29, 31]. Logistical issues, such as a lack of clear exercise integration into care, time constraints, and the need to balance treatment schedules, further limited participation [29, 34]. Accordingly, future studies should prioritise examining how specific intervention characteristics influence exercise feasibility within brain tumour populations, rather than treating feasibility as a uniform outcome.

Exercise motivations in this population appear to be strongly influenced by relational support, structural enablers, and personal goals. Encouragement from carers and healthcare providers may be essential for initiating and maintaining exercise adherence, given the high prevalence of cognitive impairment and dependence associated with disease progression, underscoring the importance of relatedness [29, 30]. Program accessibility, such as flexible scheduling, low or no‐cost sessions, and embedded referral pathways, may also enhance motivation [29]. High prevalence of unmet supportive care needs in brain tumour populations may increase levels of psychological distress and demand for services [45]. Framing findings within this theoretical context clarifies individuals' motivations for exercise participation and highlights key facilitators that inform the design of theory‐informed interventions for brain tumour populations.

The perceived physical and psychological benefits associated with exercise participation may counteract the functional decline and loss of autonomy commonly experienced following diagnosis. Physical benefits included improvements in body composition, energy levels, overall fitness, physical functioning, and treatment tolerance [29, 30]. Psychological benefits encompassed enhanced emotional well‐being, increased self‐efficacy, a greater sense of control, and reduced negative thinking [33, 34]. Additionally, participants also valued programs that fostered a sense of community or shared experience, highlighting the social benefits of group‐based exercise [29]. Specifically, supervised group‐based exercise interventions have historically been effective in broader cancer populations, improving motivation and allowing for educational components that promote safe and sustainable engagement [46]. As such, similar approaches may be both feasible and beneficial in producing comparable results for individuals with brain tumours.

### 4.1 Strengths and Limitations

To our knowledge, this is the first review to examine qualitative and quantitative data on patient perspectives of exercise engagement among adults diagnosed with brain tumours. Use of the MMAT enabled consistent evaluation of study quality across diverse methodological designs. Nonetheless, several limitations must be acknowledged. The small number of studies included in this review limits the breadth of available evidence and the strength of conclusions that can be drawn. The modest sample sizes, high attrition rates, and methodological heterogeneity limit the generalisability of findings. In addition, the outcomes assessed and reported varied across studies, making it difficult to interpret and apply findings to different tumour subtypes or among individuals with differing disease progression. This review examined contemporary evidence from the last 10 years, and the exclusion of non‐English publications may reduce generalisability to other cultural contexts. The included studies also stemmed from high‐income countries and possibly lacked diversity from different ethnic groups.

When interpreting these findings, it is important to consider that while all qualitative studies were assessed as high quality, two of the quantitative descriptive studies [31, 32] demonstrated risks of non‐response bias, limiting confidence in how well their findings reflect the wider brain tumour population. Low recruitment across included studies may limit the transferability of findings, as participants were likely those already motivated to exercise. Taken together, these limitations suggest that the strength of evidence from these studies remains preliminary, highlighting the need for more robust, well‐reported research to enhance the generalisability of the evidence base in this field.

### 4.2 Implications for Research and Practice

This review highlights several key considerations for the design and implementation of future exercise interventions for individuals with brain tumours. Programming should prioritise flexibility in delivery modes, individualisation based on individuals' preferences, and adaptability in response to fluctuations in physical and cognitive status. Specifically, to support uptake and adherence, exercise should be integrated into a holistic treatment plan through clear referral pathways from general practitioners and specialists and supported by interdisciplinary communication to ensure continuity of care. Participants consistently emphasised the motivational role of both carers and healthcare providers. Healthcare providers have a responsibility to address physical activity, and previous research has demonstrated that individuals with brain tumours expressed relief in participating in exercise programs recommended by their healthcare providers [47]. Therefore, involving carers, offering flexible scheduling, and ensuring cost‐effective access may enhance the effectiveness and sustainability of future interventions.

Future research in larger, more diverse samples should further explore how patient‐reported preferences, barriers, motivators, facilitators, and perceived benefits influence engagement and adherence across the disease trajectory. Particular attention should be given to evaluating under‐researched areas, such as resistance training preferences and outcomes for individuals with brain tumours. However, due to the challenges associated with brain tumours, tailored strategies should be incorporated to reduce barriers and maintain engagement. Consequently, alternative trial designs should also be considered for participants with poorer prognoses to generate robust evidence while accommodating the complex realities of this population. These findings could inform the development of stage‐specific guidelines and support policies that address systemic barriers and promote equitable access to tailored exercise support in neuro‐oncology.

## 5 Conclusion

The findings of this review highlight the importance of flexible, accessible, and well‐supported exercise programming tailored to the unique and variable needs of individuals with brain tumours. While home‐based and aerobic‐focused interventions show promise, further research is warranted to explore resistance training, address barriers across disease stages, and strengthen systemic support through policy and referral pathways.

## Author Contributions

**Georgia K. B. Halkett:** writing – review and editing, methodology. **Justin J. Chapman:** investigation, supervision, conceptualization, methodology, writing – review and editing. **Max E. Walker:** conceptualization, methodology, data curation, investigation, project administration, writing – original draft, writing – review and editing. **Meg A. Doohan:** conceptualisation, data curation, investigation, methodology, writing – review and editing. **David Mizrahi:** methodology, writing – review and editing. **Lisa Simmons:** methodology, writing – review and editing. **Caroline V. Robertson:** writing – review and editing.

## Funding

The authors have nothing to report.

## Ethics Statement

The protocol for this systematic review was registered with PROSPERO (ID Number: CRD420251037673). Ethical approval was not required due to this review presenting secondary data from published sources.

## Conflicts of Interest

The authors declare no conflicts of interest.

## Supporting information


**Figure S1:** PRISMA diagram detailing the process of record screening.

**Table S1:** Search terms used in search strategy. **Table S2:** Demographic Characteristics of the *N* = 7 included studies. **Table S3:** Quality Assessment of included articles using MMAT [27]. **Table S4:** Summary of exercise preferences, barriers, motivators, facilitators, and perceived benefits. **Table S5:** Summary of exercise barriers and facilitators reported across included studies by cancer stage.

## Data Availability

Data sharing not applicable to this article as no datasets were generated or analysed during the current study.
