# Supplementary material for: Exercise Preferences, Barriers, Motivators, Facilitators, and Perceived Benefits in Adults With Brain Tumours—A Systematic Review
Source: Cancer Med. 2026 Mar 31;15(4):e71731. doi: 10.1002/cam4.71731 (PMC13140841; doi:10.1002/cam4.71731)
Supplement: Supplementary file 1 — Figure S1: PRISMA diagram detailing the process of record screening. [file CAM4-15-e71731-s001.docx]

**Supplementary Materials – Figures**

**Identification**

Search Results: 6321

Duplicate records removed (*n* = 1870)

Title and Abstract Screening

(*n* = 4451)

Records excluded

(*n* = 4426)

**Screening**

Full Text Screening

(n = 25)

Reports excluded: *n* = 18

Wrong outcomes (*n* = 6)

Conference proceedings (*n* = 8)

Wrong population (*n* = 4)

Studies included in review

(*n* = 7)

**Included**

*Figure 1. PRISMA diagram detailing process of record screening.*
