# Supplementary material for: Exercise Preferences, Barriers, Motivators, Facilitators, and Perceived Benefits in Adults With Brain Tumours—A Systematic Review
Source: Cancer Med. 2026 Mar 31;15(4):e71731. doi: 10.1002/cam4.71731 (PMC13140841; doi:10.1002/cam4.71731)
Supplement: Supplementary file 2 — Table S1: Search terms used in search strategy. Table S2: Demographic Characteristics of the N = 7 included studies. Table S3: Quality Assessment of included articles using MMAT [27]. Table S4: Summary of exercise preferences, barriers, motivators, facilitators, and perceived benefits. Table S5: Summary of exercise barriers and facilitators reported across included studies by cancer stage. [file CAM4-15-e71731-s002.docx]

**Supplementary Materials - Tables**

*Table 1. Search terms used in search strategy.*

| **Search Terms** |
| --- |
| **Exercise** |
| exercis* OR “physical activit*” OR sport* OR gym* OR fitness* OR “exercise, physical” OR “exercises, physical” OR “physicalphysical exercises” OR “exercise, aerobic” OR “aerobicaerobic exercises” OR “exercises, aerobic” OR “exercise, isometric” OR “exercises, isometric” OR “isometric exercises” OR “isometricacuteacute exercises” OR “exercise, acute” OR “exercises, acute” OR “exercise training” OR “exercise trainings” OR “training,trainings,physical activity” OR “activities, physical” OR “activity, physical” OR “biometric exercise” OR effort OR “exercise capacity” OR “exercise performance” OR exertion OR “fitness training” OR “fitness workout” OR “physical conditioning, human” OR “physical effort” OR “physical exercise” OR “physical exertion” OR “physical work-out" OR “physical workout” |
| **Brain Neoplasms** |
| “brain cancer*” OR “brain neoplasm*” OR “brain metastases” OR “brain tumor*” OR “brain tumour*” OR glioma* OR “neuro-oncology” OR “brain malignant tumor*” OR “brain malignant tumour*” OR “carcinoma, brain” OR “carcinoma, cerebral” OR “cerebral carcinoma” OR “cerebral neoplasm*” or “brain carcinoma” OR “neoplasm, brain” OR “tumor, brain” OR “neoplasms, brain” OR “neoplasms, brain, malignant” OR “brain neoplasms, malignant” OR “brain neoplasm, malignant” OR “malignant cancer of brain” OR “cancer of the brain” OR “malignant neoplasms, brain” OR “malignant primary brain tumors” OR “brain neoplasms, malignant, primary” OR “brain neoplasms, primary malignant” OR “malignant primaryprimary malignantprimary malignant brain tumors” OR “neoplasms, intracranial” OR “intracranial neoplasm” OR “neoplasm, intracranial” OR “intracranial neoplasms” OR “benign neoplasms, brain” OR “benign neoplasm, brain” OR “brain benign neoplasm” OR “brain benign neoplasms” OR “brain neoplasms, benign” OR “benign brain neoplasm” OR “benignbrain neoplasm, benign” OR “neoplasms, brain, benign” OR “brain tumor, primary” OR “primary brain tumor” OR “primary brain tumors” OR “primarybrain neoplasms, primary” OR “primary brain neoplasm” OR “brain neoplasm, primary” OR “neoplasms, brain, primary” OR “brain tumor, recurrent” OR “brain tumors, recurrent” OR “recurrent brain tumor” OR “recurrent brain tumors” OR “brain metastase” |

*Table 2. Demographic Characteristics of N = 7 included studies.*

| Authors | Country | Study design | Sample type | Sample size | Dyads | Age (years) (M) ± (SD)/ Age range (years) | Diagnosis | Treatment stage | | | Active treatment | Time since diagnosis (months) ± SD |
| --- | --- | --- | --- | --- | --- | --- | --- | --- | --- | --- | --- | --- |
| Culos-Reed et al. (2017) | Canada | Quantitative non-randomised | N/a | 16 | N/a | 50.6 ± 3.7 | Glioma | Post-surgical.  Undergoing treatment | | | N/a | N/a |
| Daun et al. (2025) | Canada | Qualitative | Participants | 28 | 8 | 51.4 ± 11.7 | Glioblastoma (9); oligodendroglioma (3); astrocytoma (5); meningioma (4); germinoma (1); medulloblastoma (10); presumed glioma (1); malignant glioma not otherwise specified (1); craniopharyngioma (2); pineocytoma (1) | Any | | | Pre-treatment (1), Yes (11), No (16) | 4.9 ± 7.0 |
|  |  |  | Carers | 9 |  |  |  |  |  |  |  |  |
| Fahrenholtz et al. (2019) | Denmark | Qualitative | N/a | 5 | N/a | 30-79 | Glioma | Newly diagnosed | | | N/a | N/a |
| Gehring et al. (2018) | Netherlands | Quantitative randomised controlled trial | Intervention | 23 | N/a | 48 ± 9.4 | Astrocytoma (8); oligodendroglioma (12); oligoastrocytoma (3) | Stable grade II and III gliomas |  | No | | 7.6 ± 4.9 |
|  |  |  | Control | 11 | N/a | 48 ± 11.9 | Astrocytoma (5); oligodendroglioma (5); oligoastrocytoma (1) |  |  |  |  | 8.5 ± 8.6 |
| Halkett et al. (2021) | Australia | Qualitative | Participants | 19 | 14 | 53.4 ± 11.8 | Glioblastoma | Pre chemotherapy | | | Yes | N/a |
|  |  |  | Carers | 15 |  |  |  |  |  |  |  |  |
| Lowe et al. (2016) | Canada | Quantitative descriptive | N/a | 31 | N/a | 63.5 ± 10.4 | Brain metastases | Undergoing palliative whole brain radiotherapy | | | Yes | N/a |
| Piil et al. (2018) | Denmark | Mixed methods | Participants | 30 | N/a | 57.9/ 29-79 | Glioblastoma multiforme (23); primitive neuroectodermal tumour (1); gliosarcoma (1); anaplastic astrocytoma (4); anaplastic oligodendroglioma (1) | Newly diagnosed | | | N/a | N/a |
|  |  |  | Carers | 33 |  |  |  |  |  |  |  |  |

*Footnotes: Dyads – Responses given by a group of two individuals such as a carer and patient.*

*Table 3. Quality Assessment of included articles using MMAT* (Hong et al., 2018)

| Qualitative Studies | | | | | | |
| --- | --- | --- | --- | --- | --- | --- |
| Author (date) | **Design** | **1.1. Qualitative approach appropriate for the research question?** | **1.2. Qualitative methods adequate for the research question?** | **1.3. Are the findings adequately derived from the data?** | **1.4. Is the interpretation of results sufficiently substantiated by data?** | **1.5. Is there coherence between qualitative data sources, collection, analysis and interpretation?** |
| Daun et al. (2025) | Qualitative | Yes | Yes | Yes | Yes | Yes |
| Fahrenholtz et al. (2019) | Qualitative | Yes | Yes | Yes | Yes | Yes |
| Halkett et al. (2020) | Qualitative | Yes | Yes | Yes | Yes | Yes |
| Piil et al. (2018) | Mixed methods | Yes | Yes | Yes | Yes | Yes |
| Quantitative Descriptive Studies | | | | | | |
| Author (date) | **Design** | **4.1. Is the sampling strategy relevant to address the research question?** | **4.2. Is the sample representative of the target population?** | **4.3. Are the measurements appropriate?** | **4.4. Is the risk of non-response bias low?** | **4.5. Is the statistical analysis appropriate to answer the research question?** |
| Culos-Reed et al. (2017) | Quantitative non-randomised | Yes | Yes | Yes | Yes | Yes |
| Gehring et al. (2018) | Quantitative randomised controlled trial | Yes | Yes | Yes | Can’t tell | Yes |
| Lowe et al. (2016) | Quantitative descriptive | Yes | Yes | Yes | Can’t tell | Yes |

*Table 4. Summary of exercise preferences, barriers, motivators, facilitators and perceived benefits.*

| Authors | Preferences | Barriers | Motivators and Facilitators | Perceived Benefits |
| --- | --- | --- | --- | --- |
| Culos-Reed et al. (2017)  (N = 16) | 56.3% preferred to exercise during treatment  43.8% preferred to exercise alone  71.4% preferred to exercise unsupervised  42.9% preferred to exercise at home  86.7% preferred moderate intensity exercise intensity  56.3% preferred walking as an exercise type  100%preferred recreational over competitive exercise | N/a | N/a | N/a |
| Daun et al. (2025)  (N = 37) | Having a choice for format of delivery (i.e. online, in person or hybrid and one-on-one or group sessions)  Choice of number and intensity of sessions per week  Choice of type of exercises performed  Length and intensity of sessions varied based on treatment stage, acute energy and fatigue levels, cancer-related symptoms and goals  Sessions ranging from 15-90 min in duration  Many preferred one-on-one sessions (individualisation of sessions)  Multiple locations | Loss of independence  Loss of control  Challenges receiving information  Completing certain assessments | Improvement in physical health (physical fitness, body composition)  Improvement in psychological health (wellbeing, emotional wellness, sense of control, confidence)  Increased physical activity levels  Improvement in activities of daily living (functioning, energy levels, quality of life)  Catalyst for other health habits (improved sleep and nutrition, increased engagement in other activities)  Community-building (shared experience, positive reinforcement, protected space to share thoughts and challenges) | Increased motivation from carers and health providers  Timely referral to the program  Ability to exercise at preferred times  Enhanced support from carers  Reduced financial burden  Sense of inspiring others |
| Fahrenholtz et al. (2019)  (N = 5) | Both group based and individual training are acceptable modes of exercise | N/a | Improvement in physical health  Improvement in ADLs (increased functioning, perceived energy levels, quality of life)  Improvement in psychological health (increased motivation, hope, well-being, joy, reduced negative thoughts)  Improvement in treatment tolerance  Improvement in sleep | Training agreement  Appointments  Competitive environment  Being pushed during training  Personal goals  Relationship with healthcare professionals |
| Gehring et al. (2018)  (N = 23) | Most participants (15) chose a combination of activities.  Other participants chose a single type of exercise: indoor cycling (3); outdoor cycling (3); running/ walking (1); and swimming (1) | Lack of time  Fatigue and fear of epileptic seizures  Lack of self-discipline for home-based exercise  Lack of motivation due to divorce  Medical advice not to swim due to ear problems | N/a | N/a |
| Halkett et al. (2020)  (N = 20) | N/a | Managing symptoms while participating in program  Juggling treatment and exercise  Difficulties engaging with the program | Having an individually tailored exercise program designed by an exercise specialist  Improvements in health (Improvements in physical health & psychological health)  Regaining a sense of control  Interacting with people  Keeping active  Benefits for carers | N/a |
| Lowe et al. (2016)  (N = 31) | 65% of participants reported they were not interested in a physical activity program at the start of palliative whole brain radiotherapy  58% of participants felt they were not physically able to take part in a physical activity program  Among those open to participation, 29% preferred to exercise with family or friends  19% preferred to exercise with a spouse or caregiver.  58% preferred to exercise at home  52% preferred to exercise in the morning* 36% preferred physical activity sessions lasting 20-30 minutes, two to three times per week.  48% identified walking as the type of physical activity they were most interested in | Symptom management | 10% viewed it as a way of “being healthy.” | N/a |
| Piil et al. (2018)  (N = 63) | N/a | Progressive neurological deterioration led to significant functional and cognitive decline, making physical activity more difficult over time.  Participants experienced a reduction in daily activity capabilities and required lifestyle adjustments to accommodate their limitations. | Participants and caregivers saw physical activity as a way to actively engage in health promotion and improve well-being.  Participation in health-promoting activities was associated with maintaining hope and exerting a sense of control during treatment.  Exercise was perceived as contributing to an improved quality of life, especially when survival expectations shifted. | Hope was a central motivator  Participants were motivated to take an active role in their care, especially during early stages of treatment.  The desire to maintain quality of life became a stronger motivator as the disease progressed and survival became less certain. |

*Table 5. Summary of exercise barriers and facilitators reported across included studies by cancer stage.*

| Barrier / Facilitator | Pre-treatment | During Treatment | Post-treatment / Survivorship | Palliative | Notes / Examples |
| --- | --- | --- | --- | --- | --- |
| Symptom burden / disease progression | ✓ | ✓ | ✓ | ✓ | Physical & cognitive decline; ear issues prevented swimming (Halkett et al., 2021; Piil et al., 2018) |
| Treatment side effects |  | ✓ |  | ✓ | Fatigue, pain, side effects interfere with exercise (Lowe et al., 2016) |
| Reduced motivation / self-discipline |  | ✓ | ✓ | ✓ | Psychological barriers, fear of adverse events, perceived loss of independence (Daun et al., 2025; Gehring et al., 2018) |
| Fear of adverse events (e.g., seizures) |  | ✓ | ✓ | ✓ | Participants cautious about exercise safety |
| Access / logistics | ✓ | ✓ | ✓ |  | Difficulty with transport, program costs, scheduling conflicts (Daun et al., 2025; Halkett et al., 2021) |
| Carer / healthcare provider support | ✓ | ✓ | ✓ | ✓ | Encourages initiation and adherence (Daun et al., 2025; Fahrenholtz et al., 2019) |
|  |  |  |  |  |  |
| Structural features |  | ✓ | ✓ |  | Flexibility, scheduled appointments, low-cost/free programs |
| Intrinsic motivation / personal goals | ✓ | ✓ | ✓ |  | Quality of life, competitiveness, staying active, inspiring others (Fahrenholtz et al., 2019; Piil et al., 2018) |
